# Growth of Ceria Nano-Islands on a Stepped Au(788) Surface

**DOI:** 10.3390/ma8085205

**Published:** 2015-08-11

**Authors:** Teng Ma, Svetlozar Surnev, Falko P. Netzer

**Affiliations:** 1 College of Science, Shenyang Agricultural University, Shenyang 110168, China; E-Mail: tengma77@gmail.com; 2 Surface and Interface Physics, Institute of Physics, Karl-Franzens University Graz, A-8010 Graz, Austria; E-Mail: svetlozar.surnev@uni-graz.at

**Keywords:** ceria, stepped surfaces, gold, scanning tunneling microscopy (STM), low-energy electron diffraction (LEED), inverse catalysts

## Abstract

The growth morphology and structure of ceria nano-islands on a stepped Au(788) surface has been investigated by scanning tunneling microscopy (STM) and low-energy electron diffraction (LEED). Within the concept of physical vapor deposition, different kinetic routes have been employed to design ceria-Au inverse model catalysts with different ceria nanoparticle shapes and arrangements. A two-dimensional superlattice of ceria nano-islands with a relatively narrow size distribution (5 ± 2 nm^2^) has been generated on the Au(788) surface by the postoxidation method. This reflects the periodic anisotropy of the template surface and has been ascribed to the pinning of ceria clusters and thus nucleation on the fcc domains of the herringbone reconstruction on the Au terraces. In contrast, the reactive evaporation method yields ceria islands elongated in [01-1] direction, *i.e.*, parallel to the step edges, with high aspect ratios (~6). Diffusion along the Au step edges of ceria clusters and their limited step crossing in conjunction with a growth front perpendicular to the step edges is tentatively proposed to control the ceria growth under reactive evaporation conditions. Both deposition recipes generate two-dimensional islands of CeO_2_(111)-type O–Ce–O single and double trilayer structures for submonolayer coverages.

## 1. Introduction

Ceria is a very versatile material with applications in many different fields of catalysis, such as in the three-way automotive converter, in high-temperature solid oxide fuel cells and in a variety of catalytic reactions including water-gas shift, steam reforming of oxygenates, soot oxidation and preferential oxidation of CO [1,2,3,4,5,6]. In all these applications, the redox capabilities of ceria and its related ability to act as an oxygen storage/release agent are at the root of its successful catalytic performance. Our interest in the combination of ceria with gold surfaces is motivated by the very high activity reported recently in the low-temperature oxidation of CO [7] and in the water-gas shift reaction [8,9]. The reactivity of Au-ceria catalyst systems has been found to depend critically on particle size and shapes, with nanoscale size dimensions being particularly active [3,8,9]. The important role of the metal-oxide interface in key steps of the catalytic reaction has also been recognized [8,10]. In this context, the concept of the “inverse catalyst” as a model system, *i.e.,* a metal single crystal surface decorated by oxide nanoparticles [11,12], has been shown to be a very useful approach to study the reaction steps at the metal-oxide interface and at the oxide island boundary region with atomic and molecular precision [13,14]. Here, we report the design of an inverse catalyst system comprising ceria nanostructures with different shapes on a patterned Au single crystal surface, namely the stepped Au(788) surface, as a model platform for further catalytic studies.

The growth of ultrathin films and nanostructures of ceria on various metal single crystal surfaces has been reported in the literature, such as on Pd(111) [15], Ru(0001) [16,17,18], Pt(111) [19,20], Rh(111) [21], Au(111) [22], and Cu(111) [23,24] surfaces. Interestingly, despite the large lattice mismatch between cerium oxide and these metal substrates epitaxial growth has been observed in all cases, although in the case of continuous films a variable morphological quality has been obtained, with film roughness scaling with the lattice mismatch [25]. It has been conjectured that this heteroepitaxial growth phenomenon of ceria overlayers may be mediated by high-order coincidence lattices at the interfaces [26].

**Figure 1:**
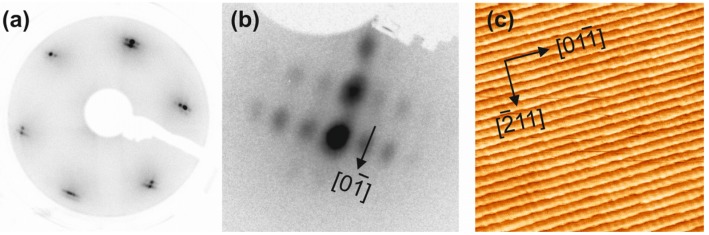
The clean Au(788) surface: (**a**) Low-energy electron diffraction (LEED) pattern, electron energy *E*_p_ = 120 eV. (**b**) LEED pattern in off-normal incidence geometry, around a first order reflection, *E*_p_ = 21 eV. (**c**) Scanning tunneling microscopy (STM) overview image (100 × 100 nm^2^; sample bias *V*_S_ = 2.2 V; tunneling current *I*_T_ = 14.7 pA).

As the shape of ceria nanoparticles matters for their catalytic activity [3,8], it is desirable to achieve some design control in the fabrication process. For model studies with targeted atomic control in the preparation and characterization process, directed assembly via physical vapor deposition (PVD) on a suitable substrate in ultrahigh vacuum is the method of choice for the growth of oxide nanostructures. Using such a bottom-up approach, the growth of ordered arrays of size-selected oxide nanoparticles has recently been achieved on an ultrathin nanopatterned aluminum oxide template in a multistep preparation procedure [27]. In the present work, we have employed a somewhat simpler procedure and have used a stepped Au(788) surface as the substrate to fabricate an inverse ceria-Au model catalyst. The Au(788) surface, a vicinal to Au(111), consists of (111) terraces, 16 atom-rows wide (3.83 nm), which are separated by [111]-type microfacets of monoatomic height (0.235 nm) [28] (see Figure 1 below). The (111) terraces display the well-known (22 × $\sqrt{3}$ ) herringbone reconstruction, which presents stacks of fcc and hcp domains separated by discommensuration lines; the latter run perpendicular to the step edges. The Au(788) surface is nanoscopically patterned in two dimensions: in [-211] direction, the step-terrace arrangement provides a structure of ~3.8 nm periodicity, whereas in [01-1] direction (*i.e.*, parallel to the step edges) the herringbone reconstruction creates anisotropic in-plane behavior with ~7.2 nm periodic distance. The Au(788) surface is relatively stable [28], and consequently has been used as a convenient template to grow arrays of metal nanoparticles [29,30], molecules [31], and metallic nanowires [32].

Here we have employed two different kinetic routes in PVD to direct the growth of ceria on Au(788) into different island arrangements and shapes: “postoxidation” and “reactive evaporation” recipes have been applied with variable oxygen pressure and temperature treatments. Surface morphology and island structures have been characterized by scanning tunneling microscopy (STM) and low-energy electron diffraction (LEED). We found that the postoxidation procedures create ordered arrays of ceria nano-islands as determined by the periodicities of the Au(788) substrate, but that the reactive evaporation procedure forms elongated islands on the terraces, with high aspect ratios and preferential growth along the [01-1] direction. The ceria islands grow essentially two-dimensional for submonolayer coverages; annealing at higher temperature leads to island growth via ripening and to ordered hexagonal CeO_2_ (111)-like structures.

## 2. Results and Discussion

Figure 1 shows LEED and STM of the clean and well-ordered Au(788) surface: the LEED pattern in Figure 1a reveals the hexagonal reflections of the (111)-type terraces, which are split both due to the step-terrace periodicity along the [-211] direction and due to the (22 × $\sqrt{3}$) reconstruction in the orthogonal [01-1] direction (see Figure 1b). These spot splittings confirm the good long range order quality of the surface, which is also recognized from the regular step-terrace arrangement in the STM image of Figure 1c.

### 2.1. Ceria on Au(788) by Postoxidation

**Figure 2:**
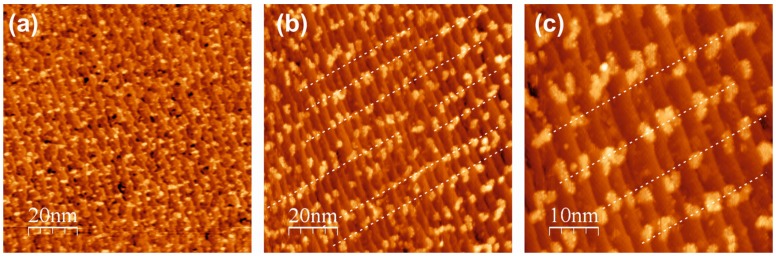
STM images of 0.14 ML Ce metal on Au(788), deposited at room temperature (**a**), and after postoxidation at 473 K in 5 × 10^−7^ mbar O_2_ (**b**,**c**). (**a**) (100 × 100) nm^2^; *V*_S_ = 2.4 V; *I*_T_ = 6.5 pA. (**b**) (100 × 100) nm^2^; *V*_S_ = 2.2 V; *I*_T_ = 14.1 pA. (**c**) (50 × 50) nm^2^; *V*_S_ = 2.2 V; *I*_T_ = 14.1 pA. The thin dashed lines in b,c are guides to the eye.

Figure 2 shows STM images of the Au(788) surface after deposition of 0.14 monolayer (ML) Ce at 300 K (a), and after postoxidation of the Ce deposits at 473 K in 5 × 10^−7^ mbar O_2_ (b,c). The cerium metal covered surface shows irregular islands in a random distribution (Figure 2a); the STM image shown is somewhat blurred; however, sharper images could not be obtained due to frequent tip changes during scanning, most probably caused by the pick-up and release of Ce adatoms. After postoxidation, the situation is significantly changed (Figure 2b,c): the oxidized ceria islands are now distributed in a more regular fashion, mostly arranged at terrace locations along lines perpendicular to the Au step edges (the thin dotted lines on the STM images serve as a guide to the eye); also, the STM imaging improved and sharper images could be measured. The ceria islands display random shapes but are located preferentially on the fcc domains of the herringbone reconstruction at the terraces, which run perpendicular to the step edges. The fcc domains show up with a brighter contrast in the STM images.

**Figure 3:**
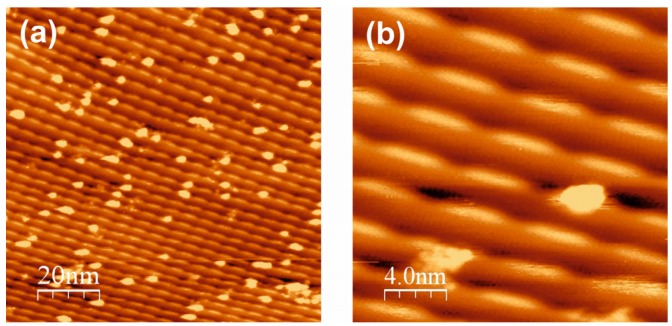
STM images of 0.07 ML of ceria on Au(788), deposited at 623 K and postoxidized at 473 K in 5 × 10^−7^ mbar O_2_. (**a**) (100 × 100) nm^2^; *V*_S_ = 2.2 V; *I*_T_ = 14.7 pA. (**b**) (20 × 20) nm^2^; *V*_S_ = 2.2 V; *I*_T_ = 14.7 pA. Note the brighter contrast areas that are associated with the fcc domains of the herringbone reconstruction.

This is most clearly seen in the STM images of Figure 3, where the attachment of ceria nano-islands to the brighter contrast regions can be recognized. In Figure 3b, two ceria nanostructures on fcc strips are clearly visible. Note that the STM images of Figure 3 have been recorded from a surface with a reduced Ce coverage of 0.07 ML, deposited at 623 K and postoxidized at 473 K.

**Figure 4:**
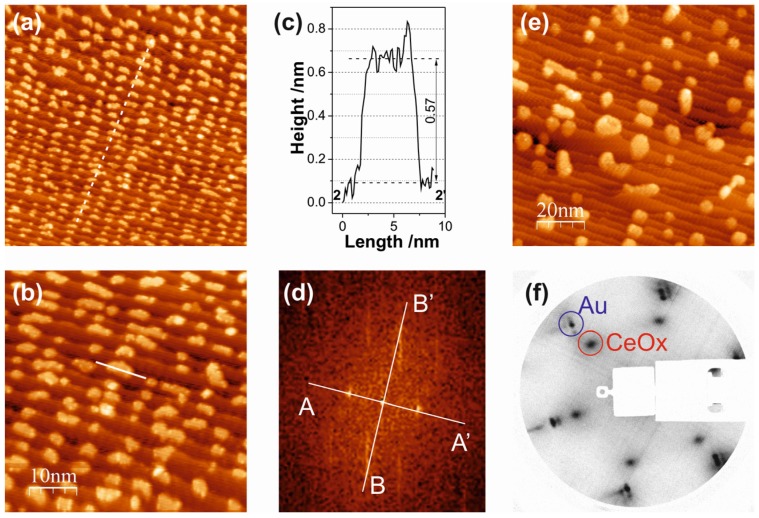
STM images of 0.14 ML ceria on Au(788), deposited at 473 K and postoxidized at 473 K in 5 × 10^−7^ mbar O_2_ (**a**) (100 × 100) nm^2^; *V*_S_ = 2.2 V; *I*_T_ = 22.1 pA. (**b**) (50 × 50) nm^2^; *V*_S_ = 2.2 V; *I*_T_ = 22.1 pA). (**c**) Line scan along the solid line in the STM image (**b**). (**d**) Fast Fourier transform (FFT) representation of the STM image (**a**); A-A´and B-B´ correspond to the directions parallel and perpendicular to the steps, respectively. (**e**) STM image of the surface (**a**) after annealing in 5 × 10^−7^ mbar O_2_ at 823 K (100 × 100 nm^2^; *V*_S_ = 2.0 V; *I*_T_ = 16.8 pA). (**f**) LEED pattern of the annealed surface (**e**) (*E*_P_ = 120 eV); Au substrate and ceria reflections are indicated.

The regularity of the ceria nano-island growth on fcc terrace stacks becomes improved after Ce deposition and postoxidation at 473 K (*p*(O_2_) = 5 × 10^−7^ mbar O_2_)—see Figure 4a,b. The ceria islands arranged on the fcc domain lines in [-211] direction, and the islands appear to have narrowed their size distribution (5 ± 2 nm^2^). The Ce nano-islands are typically ~0.6 nm in height, as evidenced by the STM line profile in Figure 4c. This indicates a double trilayer (O–Ce–O) geometry, as found in previous studies [26] and discussed below (note that a (111)-type (O–Ce–O) trilayer in bulk CeO_2_ measures ~0.31 nm). The fast Fourier transform (FFT) of the STM image in Figure 4a, given in Figure 4d, confirms the visual impression of the ordered arrangement of the ceria nano-islands: the sharp FFT spots along A-A´ indicate a ~6.5 nm periodicity parallel to the steps, but along B-B´, *i.e.*, perpendicular to the steps, the periodicity is more variable and measures 3.0–3.8 nm. Within the experimental error and taking into account the irregularities of the ceria nano-island shapes, this is compatible with the fcc domain and step periodicities of the Au(788) surface. The two-dimensional (2-D) patterning of the Au(788) surface thus leads to the pinning of ceria nano-islands and to the formation of a ceria nanoparticle superlattice.

Annealing of the ceria islands to 823 K in 5 × 10^−7^ mbar O_2_ leads to sintering and crystallization of the oxide phase: as seen in Figure 4e, the oxide island density is significantly reduced and the island sizes are increased. The LEED picture in Figure 4f shows the hexagonal array of reflections from the Au substrate terraces and the reflections from the so-called (1.33 × 1.33) pattern of ceria; the latter is referenced to the Au lattice and indicates the crystalline ordering within the ceria islands. The lattice constant of the ceria as estimated from the LEED pattern is ~0.384 nm, which is close to the value of 0.389 nm of bulk CeO_2_. The measured lattice constant value of 0.384 nm is, however, by itself insufficient to identify the cerium oxide phase as CeO_2_, since the lattice constant of the reduced Ce_2_O_3_ phase (0.382 nm) is also close to this value. Our own experience [21,26] as well as evidence in the literature [18,33,34,35,36] indicates, however, that, at the given oxidation conditions (5 × 10^−7^ mbar O_2_, 473 K), the oxide stoichiometry is close to CeO_2_. We thus propose that the ceria nanoparticles formed on Au(788) by postoxidation in an oxygen pressure *p*(O_2_) > 10^−7^ mbar O_2_ are CeO_2_(111)-type trilayer structures; most of the islands are double trilayers, but single trilayer islands are also occasionally observed. Note that this is in agreement with previous density functional theory (DFT) calculations, which found that single and double CeO_2_-type trilayer structures are of similar stability [26].

An important morphological aspect of the ceria growth by the postoxidation method is the pinning of the oxide nano-islands to the fcc domains of the herringbone reconstruction at the Au(788) terraces, thus creating this nanoparticle superlattice. Interestingly, the Ce metal deposits do not show this preferential arrangement—see Figure 2a. This may be due to the strong interaction of the Ce adatoms with the Au surface, as evidenced by the easy formation of a Ce-Au surface alloy [37]; this strong interaction precludes the diffusion of the Ce adatoms. After oxidation, the ceria-Au interaction becomes much weaker and small ceria clusters can diffuse to the most preferred adsorption sites, nucleating on the fcc stacks. Annealing at higher temperatures leads to ripening and cluster aggregation processes and destroys the nanoparticle superlattice—see Figure 4e.

### 2.2. Ceria on Au(788) by Reactive Evaporation

**Figure 5:**
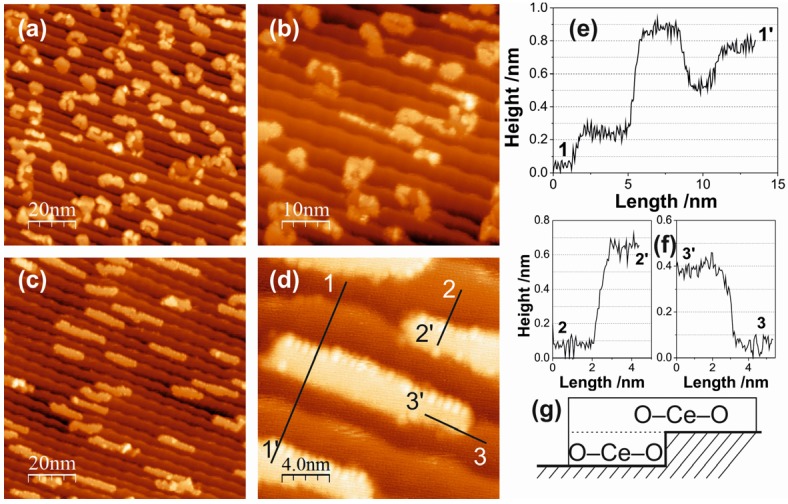
STM images of 0.14 ML ceria on Au(788): (**a**,**b**) Fabricated by reactive evaporation at 473 K in 1 × 10^−8^ mbar O_2_. (**a**) (100 × 100) nm^2^; *V*_S_ = 2.3 V; *I*_T_ = 28.9 pA. (**b**) (50 × 50) nm^2^; *V*_S_ = 1.6 V; *I*_T_ = 30.2 pA. (**c**,**d**) Fabricated by reactive evaporation at 473 K in 1 × 10^−6^ mbar O_2_. (**c**) (100 × 100) nm^2^; *V*_S_ = 2.0 V; *I*_T_ = 24.1 pA. (**d**) (50 × 50) nm^2^; *V*_S_ = 2.0 V; *I*_T_ = 24.1 pA. (**e**,**f**) Line scans along the solid lines 1-1´, 2-2´ and 3-3´ in image (**d**). (**g**) Cartoon illustrating a ceria island attached to the Au step edge, as seen in the STM image (**d**).

Deposition of Ce in 1 × 10^−8^ mbar O_2_ at 473 K substrate temperature yields ceria islands of irregular shape and randomly distributed over the stepped Au surface, as shown in the STM images of Figure 5a,b. The islands are not confined by the terraces and occasionally grow over the step edges; single layer islands and islands with a partial second layer can be distinguished. Overall, the oxide island distribution is independent of the periodicities of the Au substrate surface. The island density for 0.14 ML ceria coverage is of the order of 70 per (100 × 100) nm^2^ (Figure 5a), which is a factor of 5 lower than in the case of the postoxidation recipe (where island densities of ~350 per (100 × 100) nm^2^ for the same coverage have been estimated (e.g., from Figure 4a).

The morphology of the ceria deposits is significantly changed, if the oxygen pressure during reactive evaporation is increased: the STM images of Figure 5c,d have been recorded after Ce evaporation in 1 × 10^−6^ mbar O_2_ (473 K substrate). Here, the ceria grows in form of long and narrow islands, elongated in the [01-1] direction, *i.e.*, parallel to the step edges. A typical ceria island measures 15 nm and 2.5 nm in length and width, respectively, thus displaying an aspect ratio of ~6. The island density is further reduced to ~50/(100 × 100) nm^2^. The zoomed-in STM image of Figure 5d reveals interesting aspects of the structure of the ceria islands, which are contained in the line scans along the solid lines in the STM image in Figure 5d, given in Figure 5e,f. The line scan 1-1´ (panel e) shows that the ceria island is ~0.6 nm high from the lower Au terrace, but only ~0.35 nm from the upper terrace: this is consistent with two (O–Ce–O) trilayers at the lower terrace, but only one trilayer at the upper terrace. The line scans 2-2´ (from the island in the middle of Figure 5d) and 3-3´ confirm this picture: they measure ~0.6 nm and 0.35 nm for the heights from lower and upper terraces, respectively. The ceria islands, thus, form a double trilayer on the lower terraces, but the upper trilayer then also grows onto the upper terraces. The cartoon in Figure 5g illustrates the situation. This island structure may be the result of a reduction of the Schwoebel-Ehrlich barrier at the Au steps, once the first ceria trilayer is attached to the lower step edge.

**Figure 6:**
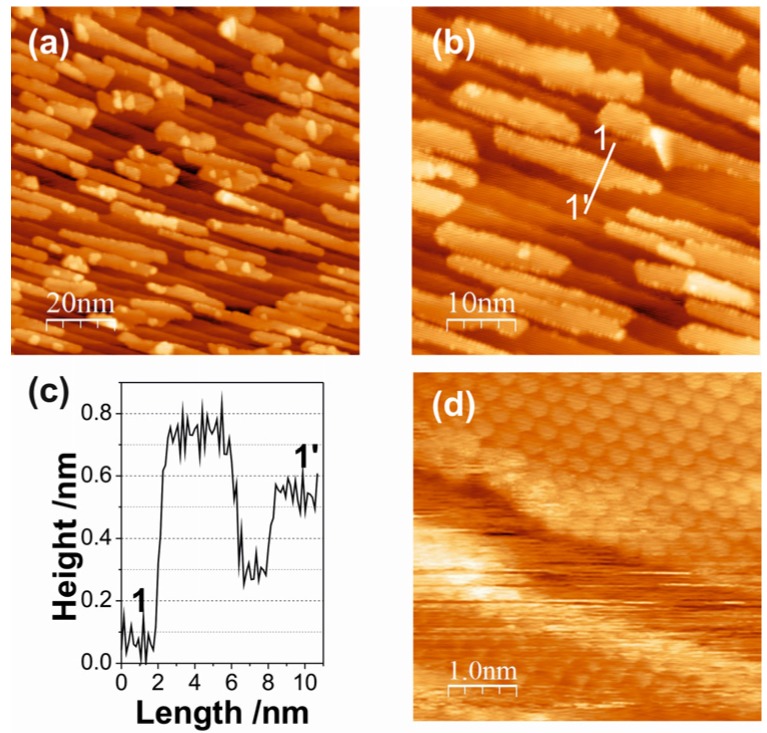
(**a**,**b**) STM images of 0.28 ML ceria on Au(788); (**a**) (100 × 100) nm^2^; *V*_S_ = 2.2 V; *I*_T_ = 22.0 pA. (**b**) (50 × 50) nm^2^; *V*_S_ = 1.7 V; *I*_T_ = 16.8 pA. (**c**) Line scan along the solid line in (**b**). (**d**) High resolution STM image of a ceria island surface ((5 × 5) nm^2^; *V*_S_ = 0.1 V; *I*_T_ = 350 pA).

Increasing the ceria coverage leads to an increase in the number of islands and to moderate increases in island size (Figure 6a,b) but to little changes in the island morphology (see the line scan 1-1´ in Figure 6c). The atomically resolved STM image in Figure 6d, taken from the top surface of an island in Figure 6b, reveals that the ceria islands are crystalline with a hexagonal lattice. This is confirmed by the LEED pattern from this surface (not shown), which contains the hexagonal reflections of the (1.33 × 1.33) structure of ceria, in accordance with the proposed CeO_2_(111)-type islands.

A growth scenario during the reactive evaporation of ceria on the Au(788) surface may be visualized as follows. At low oxygen pressure (10^−8^ mbar), the ceria deposits are not fully oxidized and the substoichiometric, defective CeO_2 − *x*_ clusters nucleate randomly on the surface due to a strong ceria-Au interaction; this results in irregular island distribution and shape (Figure 5a,b). At high oxygen pressure (10^−6^ mbar), the ceria clusters are fully oxidized to stoichiometric CeO_2_, the ceria-Au interaction is concomitantly weaker and the ceria cluster diffusion is enhanced. This diffusion is anisotropic, with preference along the [01-1] direction, leading to nucleation at the step edges. The Schwoebel-Ehrlich barrier precludes step crossing and the growth front perpendicular to the step edges creates the elongated island structures seen in the STM images of Figure 5c,d and Figure 6a,b. Note that the length of the ceria islands extends beyond the periodicity distance of the herringbone reconstruction at the Au terraces, which apparently does not pin ceria nucleation centers in the oxygen atmosphere of the reactive evaporation conditions. As mentioned above, DFT calculations [26] have indicated that single (O–Ce–O) trilayer and double trilayer islands structures on metal surfaces are close in energy. In the present case, the second (O–Ce–O) trilayer on top of the first trilayer, which is attached to the Au step edge, is observed to grow across the step edge onto the upper terrace, suggesting that the Schwoebel barrier at the steps is reduced by the attached ceria. The growth of three-dimensional ceria islands beyond double trilayer has not been observed for submonolayer coverages at the preparation conditions here explored.

## 3. Experimental Section

The experiments have been performed in a custom-designed three-chamber ultra-high vacuum (UHV) system with a base pressure of ~1–2 × 10^−10^ mbar, equipped with a LEED optics, a room temperature STM (Omicron μ-STM), sample cleaning and transfer capabilities as well as provisions for thin film evaporation [21]. The STM images have been measured in a constant current mode, with electrochemically etched W tips. The Au(788) surface has been cleaned by cycles of 1.0 keV Ar^+^ ion sputtering and annealing to 800 K. Cerium has been evaporated from metallic granulate in a tungsten crucible heated by electron beam and the evaporation rate has been monitored by a quartz microbalance: the ceria coverages quoted are in effective monolayers (ML) of cerium deposition, as referenced to the atomic density of the Au(111) surface (*i.e.*, 1.39 × 10^15^ atoms/cm^2^). Two major preparation procedures have been utilized to fabricate the cerium oxide deposits: postoxidation and reactive evaporation. The postoxidation procedure consists of the evaporation of Ce metal in UHV, at a given Au substrate temperature, followed by oxidation of the metallic deposits in O_2_ at variable pressure and elevated substrate temperature. In the reactive evaporation procedure, Ce is evaporated directly in O_2_ atmosphere at a given pressure and substrate temperature.

## 4. Conclusions

Inverse catalyst model systems of ceria on Au have been designed using physical vapor deposition recipes and different kinetic routes, resulting in the growth of ceria nanoparticles with different morphologies and shapes on a nanopatterned Au(788) surface. The so called postoxidation method, *i.e.*, the deposition of cerium metal first, followed by oxidation at various oxygen pressures and temperatures, yields ceria nano-islands with a relatively narrow size distribution arranged in a two-dimensional superlattice, which reflects the periodic anisotropy of the Au(788) template surface. This directed self-assembly is based on the preferential adsorption of small ceria clusters—formed by oxidation of the cerium metal deposits at the Au surface—on the fcc domains of the herringbone reconstruction on the Au terraces. The ceria nano-islands consist of CeO_2_(111)-type structures, mostly in the form of double O–Ce–O trilayers. The ceria nanoparticles display a different morphology if prepared by reactive evaporation. At sufficiently high oxygen pressures (≥1 × 10^−6^ mbar), islands elongated in [01-1] direction (*i.e.*, parallel to the step edges) with high aspect ratios are formed. They are CeO_2_-like trilayers as well, attached at the Au step edges, where the second O–Ce–O trilayer can grow over the step edge onto the upper terrace area. The growth mode is controlled by diffusion of small ceria clusters, formed at the arrival of Ce adatoms at the Au surface, along the step edges with a Schwoebel-Ehrlich barrier precluding step crossing for the first monolayer; pinning of ceria clusters at the herringbone reconstruction and concomitant nucleation on fcc domains appears to be inoperative under reactive evaporation conditions.

It is of interest to compare the growth of ceria nanoparticles on Au(788) with the one reported on Au(111) [22]. In both cases, CeO_2_(111)-type island structures are formed. While step edges also provide nucleation sites for ceria growth on Au(111), the herringbone reconstruction on large flat Au(111) areas does not induce ordered nano-island arrangements. The reactive deposition method for ceria coverages comparable to those reported here generates more roundish nanostructures on Au(111), in contrast to the high aspect ratio particles on the stepped Au(788) surface. Overall, the growth of ceria on Au(788) is strictly 2-D (single and double trilayer structures) for the submonolayer coverages investigated here, whereas it is more three-dimensional on Au(111) [22,8]. The templating properties of the stepped Au(788) surface thus allow us to tune the ceria island shapes and arrangement via kinetic channels and intrinsic surface barriers. In view of the shape-dependent catalytic activity of ceria nanoparticles [3,5,8], the inverse ceria-Au catalysts presented in this work provide model systems for catalytic surface reaction studies and a platform of interest for further investigations in the field of advanced nanocatalysis.
